# Plasmid-Mediated Spread of Antibiotic Resistance by Arsenic and Microplastics During Vermicomposting

**DOI:** 10.3390/antibiotics14121230

**Published:** 2025-12-06

**Authors:** Rui Xin, Huai Lin, Zijun Li, Fengxia Yang

**Affiliations:** 1 Agro-Environmental Protection Institute, Ministry of Agriculture and Rural Affairs, Tianjin 300191, China; 2 Ministry of Education Key Laboratory of Pollution Processes and Environmental Criteria, College of Environmental Science and Engineering, Nankai University, Tianjin 300071, China; 3 Chaoyang District Agricultural and Rural Comprehensive Service Center of Beijing, Beijing 100125, China

**Keywords:** antibiotic resistance genes, plasmid, vermicomposting, arsenic, polyethylene terephthalate

## Abstract

**Background:** The efficiency of vermicomposting in reducing antibiotic resistance genes (ARGs) in dairy manure may be compromised by co-pollutants like arsenic (As) and microplastics. Specifically, plasmids serving as carriers and vectors of ARGs were largely distributed in this process. However, the impact of As and microplastics on plasmids carrying ARGs during vermicomposting is largely unknown. **Methods:** This study utilized a controlled experimental design and applied plasmid metagenomics to investigate the individual and combined effects of As and polyethylene terephthalate (PET) microplastics on plasmid-mediated ARG dynamics during vermicomposting. **Results:** We found that vermicomposting alone mainly enriched non-mobilizable plasmids, while PET microplastics selectively promoted conjugative and mobilizable plasmids, whereas As significantly increased all plasmid types. Moreover, both PET or As alone and combined exposure (PET and As) increased total ARG abundance, with their combination inducing synergistic ARG enrichment despite unchanged total plasmid abundance. Furthermore, co-occurrence network analysis combined with ARGs/plasmid ratio assessments demonstrated that As influences ARGs through co-selective pressure by enriching ARGs co-localized with As resistance genes (e.g., the *ars* operon) on plasmids while simultaneously promoting horizontal gene transfer (HGT) via activation of oxidative stress and SOS response pathways. In contrast, PET primarily facilitates ARG dissemination through a “metabolism-resistance” coupling strategy by enriching colonizing bacteria with PET-degrading capacity. Their co-exposure formed As-enrichment hotspots on PET microplastic surfaces, functioning as a “super-mixer” that selectively screened for superbugs carrying potent resistance mechanisms (e.g., *bla_OXA-50_* and *mdtB*/*mdtE*). **Conclusions:** This study provides the first plasmidome-level evidence of synergistic ARG propagation by As and PET microplastics during vermicomposting, highlighting mobile genetic elements’ critical role in co-pollutant risk assessments.

## 1. Introduction

Antibiotic resistance is one of the most pressing global health challenges of the 21st century [1]. The emergence of antibiotic-resistant bacteria is largely attributed to the dissemination of antibiotic resistance genes (ARGs) through horizontal gene transfer (HGT), a process primarily mediated by plasmids [2]. Extrachromosomal genetic element plasmids can be categorized into conjugative plasmids, mobilizable non-conjugative plasmids, and non-mobilizable plasmids. Among them, mobilizable plasmids are capable of transferring between bacteria via conjugation, and they play a critical role in the spread of ARGs [3].

Soils, particularly those impacted by agricultural activities, are recognized as critical reservoirs for the emergence and dissemination of ARGs. A major source of exogenous ARGs in agricultural soils is the application of livestock and poultry manure, which exacerbated ARG contamination and spread [4]. Therefore, effective ARG reduction technologies prior to manure application are essential to curb the transmission of antibiotic resistance. Vermicomposting, a bio-oxidative process that utilizes the symbiosis between earthworms and microorganisms, has emerged as a promising and sustainable waste valorisation technology [5]. This approach not only produces nutrient-rich humus but also reduces the abundance of some ARG types during composting [6,7]. This reduction effect is primarily attributed to microbial community restructuring and antibiotic degradation induced by the feeding activities of earthworms [8]. Consequently, it is regarded as one of the most promising methods for mitigating ARGs prior to manure application on land [9].

However, contaminants present in the raw manure may significantly interfere with ARG removal during the vermicomposting process. In modern agriculture, microplastics and arsenic (As) are pollutants of growing concern as they are both abundantly present in agricultural environments [10,11]. As, a toxic metalloid derived from industrial and agricultural activities, could exert strong co-selective pressure on microbial communities [12,13]. This pressure not only enriches metal resistance genes (MRGs) but may also promote the co-selection of ARGs [14]. It is essential to clarify several key concepts in this context: “co-occurrence” describes the simultaneous presence of different genes (e.g., ARGs and MRGs) within a microbial community; “co-localization” refers to their physical presence on the same mobile genetic element (MGE), such as a plasmid; and “co-selection” specifically denotes the process by which selection for one trait (e.g., metal resistance) directly enriches a genetically linked but unselected trait (e.g., antibiotic resistance). Microplastics from these environments usually originate from plastic fragmentation and organic fertilizers, which serve as pervasive contaminants that provide unique ecological niches for microbial colonization and biofilm formation [10]. Previous studies revealed that these biofilms can act as hotspots for HGT, facilitating the exchange of MGEs such as plasmids [15,16]. Moreover, microplastic-mediated biofilm development further intensifies genetic exchange hotspots, thereby synergistically amplifying the selective pressure imposed by heavy metals [15]. The interaction between microplastics and As may thus generate synergistic effects that profoundly influence the dissemination and enrichment of ARGs in vermicomposting ecosystems. Despite this concerning possibility, the impact of their combined stress on the fate of ARGs during the vermicomposting process—particularly from the perspective of plasmid genomics—remains unexplored to date.

Moreover, most current research on the reduction in ARGs during vermicomposting has focused on bulk community dynamics using metagenomics and quantitative PCR (qPCR). These approaches cannot capture the crucial role of plasmids—autonomously mobile vectors that serve as fundamental vehicles for the rapid dissemination of ARGs across bacterial taxa [3]. Indeed, a growing body of evidence indicates that plasmid-mediated gene transfer in environmental settings has become a critical process sustaining antibiotic resistance. Environmental studies have consistently highlighted the prominence of plasmids in ARG dissemination. For instance, numerous ARG-carrying plasmids identified in environmental isolates are mobilizable or conjugative, facilitating widespread gene transfer [17,18]. Furthermore, once incorporated into broad-host-range plasmids (e.g., those belonging to incompatibility groups such as IncP and IncQ), ARGs can readily transfer across a wide phylogenetic range of bacteria, including various pathogens, directly impacting the health of receiving ecosystems and nearby human populations [19]. Thus, it is critically important to determine whether the vermicomposting process, particularly under the influence of co-existing contaminants, disrupts or inadvertently and selectively enriches these potent plasmid-induce ARG carriers.

This study employed a plasmid-centric metagenomic approach to investigate the individual and combined effects of As and PET microplastics on the plasmidome and associated ARGs during vermicomposting. A controlled experiment with five treatment groups was implemented (1) to compare the individual and combined effects of As and PET microplastics on plasmidome composition and the abundance and diversity of plasmid-associated ARGs, analyzing the differences between combined exposure and individual treatment effects; (2) to identify correlations between plasmid types and ARG profiles under different treatments; and to discuss whether potential mechanisms—such as co-selection and horizontal gene transfer—contribute to the observed ARG dynamics within the detection limits of our methodological approach.

## 2. Results

### 2.1. Effects of Different Treatments on Plasmid Dynamics During Vermicomposting

**Figure 1:**
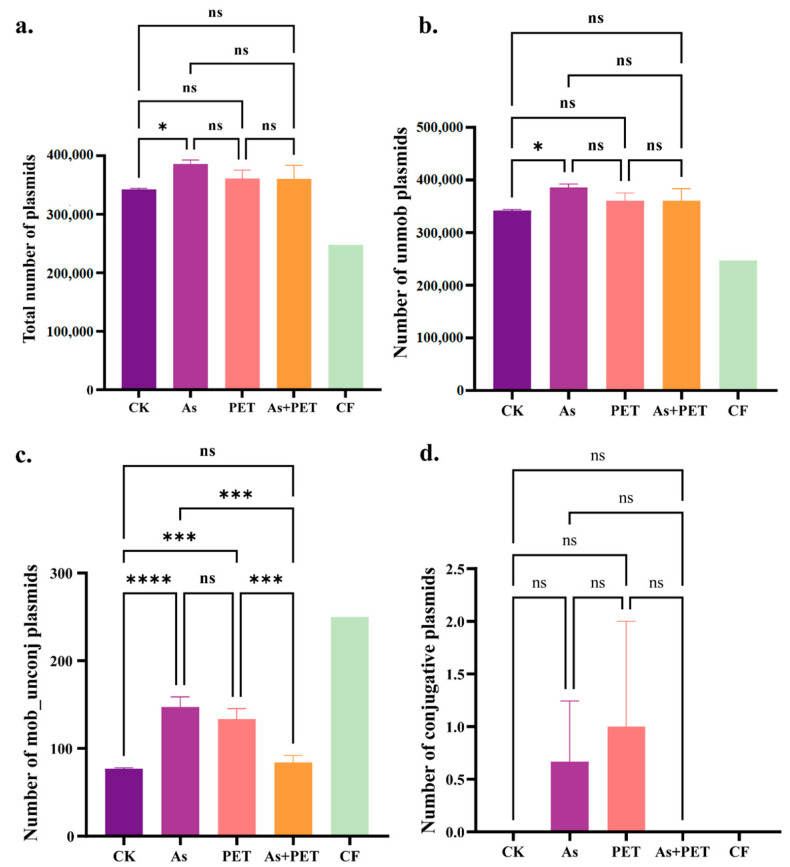
Abundance of plasmids among different groups. (**a**) Total abundance of plasmids among groups (*n* = 3). (**b**–**d**) Abundance of non-mobilizable plasmids, mobilizable plasmids, and conjugative plasmids (*n* = 3). CK represents the control group (only added earthworms without pollution exposure). As represents the Arsenium treatment (arsenium exposure after earthworms are added). PET represents the polyethylene terephthalate treatment (PET microplastic exposure after earthworms are added). AS + PET represents the arsenium and PET combined treatment (AS + PET exposure after earthworms are add), and CF represents the untreated group (original fecal matter without any added substances and treatments).*Symbols indicate statistical significance: * *p* < 0.05, *** *p* < 0.001, **** *p* < 0.0001, ns represent not significant.

Different treatments significantly altered the total plasmid abundance during the vermicomposting of dairy manure. Compared to untreated manure, vermicomposting alone increased total plasmid abundance (Figure 1a). Notably, this increase was primarily driven by non-mobilizable plasmids, whereas mobilizable but non-conjugative plasmids exhibited a declining trend (Figure 1b,c).

The addition of As during vermicomposting significantly increased the abundance of all plasmid types, including non-mobilizable, mobilizable, and conjugative plasmids (Figure 1). In contrast, while PET microplastics did not significantly alter the total plasmid content, they markedly increased the abundance of conjugative and mobilizable plasmids (Figure 1).

Interestingly, combined exposure to both contaminants showed minimal impact on total plasmid abundance during vermicomposting. Compared to the individual As or PET treatments, the co-treatment strategy neither promoted an increase in non-mobilizable plasmids nor exerted a significant stimulatory effect on mobilizable non-conjugative plasmids (Figure 1).

### 2.2. Enrichment of Plasmid-Associated ARGs by As and PET During Cattle Manure Vermicomposting

**Figure 2:**
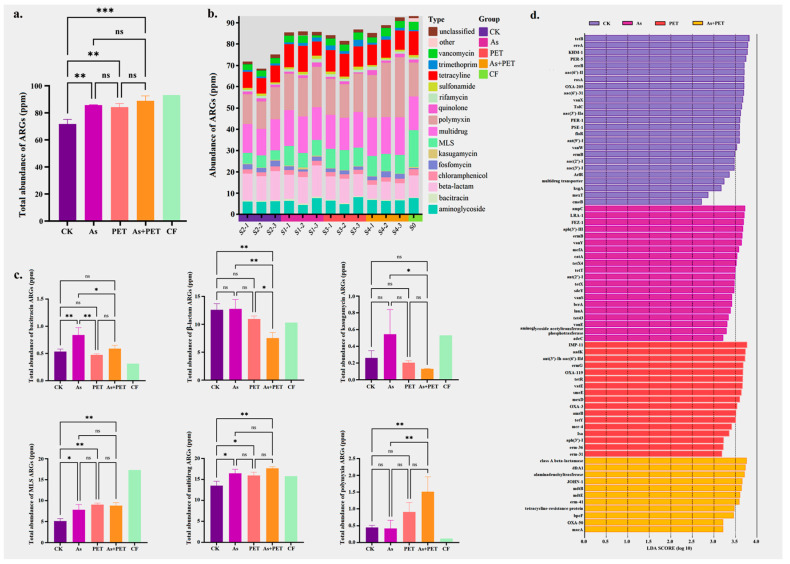
Response of different types of ARGs to different treatments. (**a**) Total abundance of ARGs among groups (*n* = 3). (**b**) Different types of ARGs among groups (*n* = 3). (**c**) Significant response types of ARGs among groups (*n* = 3). (**d**) Differential abundance analysis of ARGs identified by Linear Discriminant Analysis (LDA) effect size (LEfSe) (*n* = 3). *Symbols indicate statistical significance: * *p* < 0.05, ** *p* < 0.01, *** *p* < 0.001, ns represent not significant.

Heavy metals and microplastics promoted the enrichment of plasmid-associated ARGs during the vermicomposting of cattle manure. Specifically, treatments involving As, PET microplastics, and their combination significantly increased the abundance of plasmid-associated ARGs during vermicomposting (Figure 2a). Although not statistically significant, these treatments also tended to increase the diversity of plasmid-associated ARG types (Figure S1). Clustering of ARG profiles was separated across different treatment groups, indicating that the impact of various treatments on ARG carriage was treatment-specific (Figure 2, Figures S2 and S3).

Exposure to As resulted in a broad-spectrum increase in plasmid-associated ARG abundance, affecting 12 out of 17 major ARG types (Figure S4). Notably, the As treatment significantly enriched bacitracin, MLS, and multidrug resistance genes *(p* < 0.05; Figure 2c). In comparison, PET microplastics selectively promoted plasmid-associated ARGs associated with aminoglycoside, chloramphenicol, kasugamycin, polymyxin, rifamycin, tetracycline, trimethoprim, vancomycin, and unclassified types (Figure S4), with significant increases also observed in MLS and multidrug resistance categories (Figure 2c). Overall, As exposure exerted a substantially stronger influence on ARG increase than the PET treatment.

Although the combined As and PET treatment did not significantly alter total plasmid abundance (Figure 1), it induced marked enrichment of plasmid-associated ARGs, exceeding the levels observed in the individual treatments (albeit not statistically significant) (Figure 2a). In addition to the significant enrichment of MLS and multidrug resistance genes, the combined treatment also led to a notable increase in polymyxin resistance genes, accompanied by a significant decrease in β-lactam resistance gene abundance (Figure 2c).

At the gene-specific level, the As treatment group exhibited significant enrichment of 21 plasmid-associated ARGs, including *bla_ampC_*, *bla_LRA-1_*, *bla_FEZ-1_*, *aph(3′)-II*, *ermB*, *vanY*, *mefA*, *catA*, and *tetX4* (Figure 2d). The PET treatment group displayed enrichment of 17 plasmid-associated ARGs, with representative genes such as *bla_IMP-11_*, *aadK*, *ant(3′)-Ih-aac(6′)-Id*, *ermG*, *bla_OXA-119_*, *tetR*, *vatE*, *smeE*, *mexD*, *bla_OXA-3_*, *amrB*, and *tetY* (Figure 2d). In the combined PET and As treatment group, 11 plasmid-associated ARGs were significantly enriched, including *class A beta-lactamase*, *dfrA1*, *alanine adenosyltransferase JOHN-1*, *mdtB*, *mdtE*, and *erm-41* (Figure 2d).

**Figure 3:**
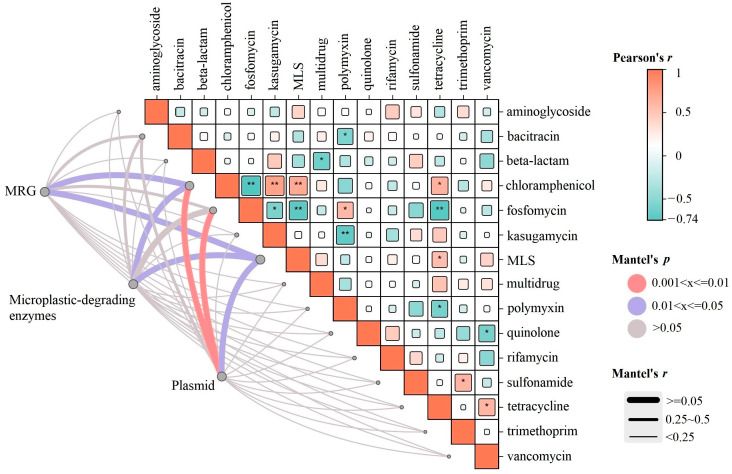
Mantel test results of ARGs with plasmids, MRGs, and microplastic-degrading enzymes. The line represents the regression fit, and the Mantel r statistic and *p*-value are displayed on the graph. Significance levels are indicated as follows: * *p* < 0.05 and ** *p* < 0.01 (*n* = 3).

Mantel tests were performed to assess the correlations between ARG profiles and the community profiles of MRGs, microplastic-degrading genes, and plasmid abundance. The results revealed distinct drivers for different ARG categories. MRGs and microplastic-degrading genes were significantly correlated with MLS (*p* < 0.05) and chloramphenicol (*p* < 0.05) resistance genes. In contrast, plasmid abundance exhibited the strongest overall association with the ARG profiles, showing significant correlations with MLS (*p* < 0.05), chloramphenicol (*p* < 0.01), and fosfomycin (*p* < 0.01) resistance genes (Figure 3).

### 2.3. Contribution of Plasmids to ARG Variation During Cattle Manure Vermicomposting

**Figure 4:**
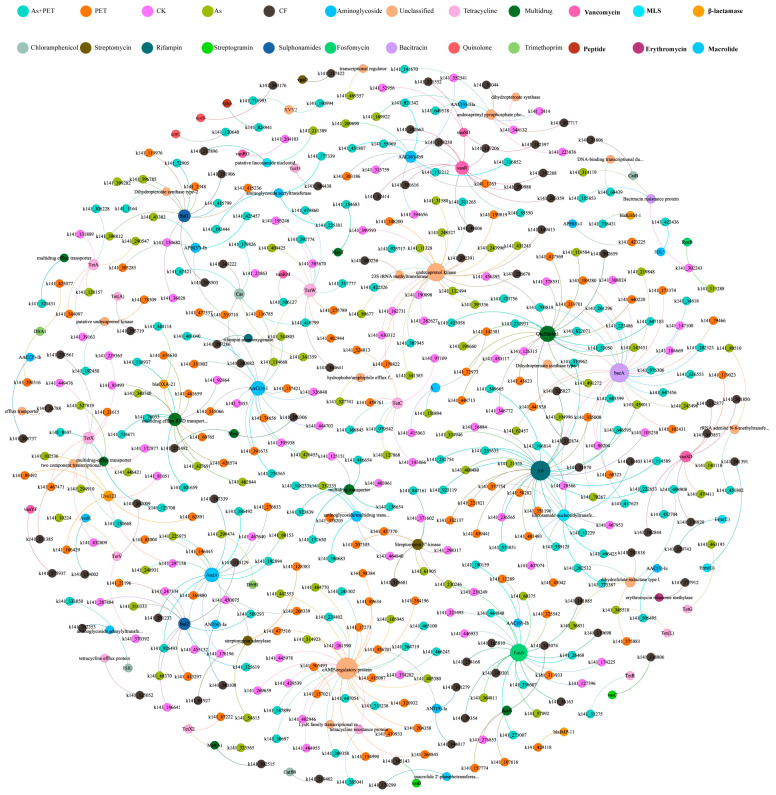
Co-occurrence network between ARGs and plasmids. Nodes represent plasmids/ARG subtypes, and they are colored according to their sample type. Connections (edges) between nodes represent positive (Spearman’s *r* > 0.6) and statistically significant (*p* < 0.01) correlations (*n* = 3). The size of each node is proportional to its relative abundance.

Plasmids are carriers of ARGs. Co-occurrence network analysis of potential ARGs from host plasmids revealed a high prevalence of aminoglycoside, multidrug, and tetracycline resistance genes (Figure 4). Although most identified plasmids were non-mobilizable, with only a minority being mobilizable but non-conjugative (Table S1), the proportion of ARG-carrying mobilizable but non-conjugative plasmids (0.5%) was substantially higher than that of ARG-carrying non-mobilizable plasmids (0.009%). Notably, tetracycline, MLS, and aminoglycoside ARGs were detected in mobilizable but non-conjugative plasmids, which may explain the high abundance of these ARG types.

The most prevalent plasmid-carried ARGs (degree ≥ 10) included the aminoglycoside resistance genes *AAC(3)-I* (degree = 15), *aac(6′)-Ib9* (degree = 9), and *AadA* (degree = 15); the rifamycin resistance gene *Arr* (degree = 28); the peptide resistance gene *bacA* (degree = 26); the phosphonic acid resistance gene *fosB* (degree = 22); the disinfectant resistance gene *QacEdelta1* (degree = 19); the glycopeptide resistance gene *vanR* (degree = 13); the tetracycline resistance gene *tet(W)* (degree = 11); and the sulfonamide resistance genes *sul1* and *sul2* (degrees = 11 and 10, respectively). Additionally, several enzymes associated with broad-spectrum drug resistance, including *cAMP-regulatory protein* (degree = 32), were widely prevalent in plasmid genomes.

**Figure 5:**
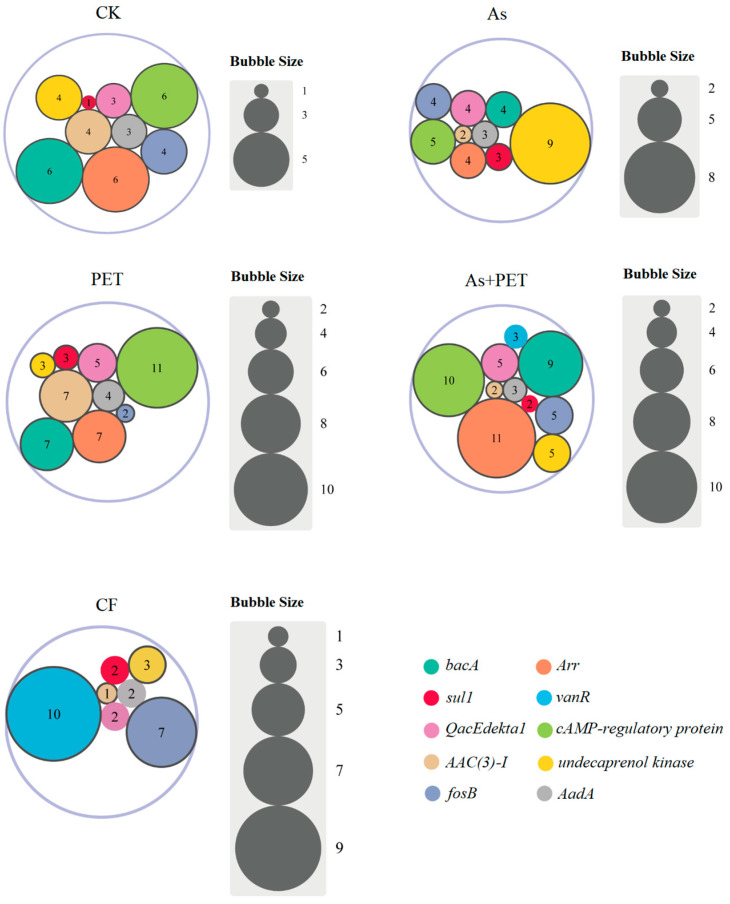
Variations in ARG abundance and diversity carried by plasmids under different contamination conditions. The bubble chart illustrates the abundance and distribution of major plasmid-borne ARGs across four treatment groups: control (CK), arsenic (As), polyethylene terephthalate (PET), combined arsenic and PET (As + PET), and untreated (CF) (*n* = 3). The bubble size corresponds to the relative abundance of each ARG normalized by plasmid copy number.

Analysis of the top 10 most abundant plasmid-associated ARGs across treatments revealed distinct compositional profiles of resistance plasmids. The As treatment significantly increased the abundance of plasmids carrying *undecaprenol kinase* (2.25-fold higher than the control) and *sul1* (3-fold higher). Under the PET treatment, plasmids carrying *cAMP-regulatory protein*, *AAC(3)-I*, and *QacEdelta1* increased by 1.83-, 1.75-, and 1.67-fold, respectively. In the combined treatment, *Arr*-, *cAMP*-, *bacA*-, *QacEdelta1*-, and *sul1*-carrying plasmids increased by 1.83-, 1.67-, 1.5-, 1.67-, and 2-fold, respectively (Figure 5).

**Figure 6:**
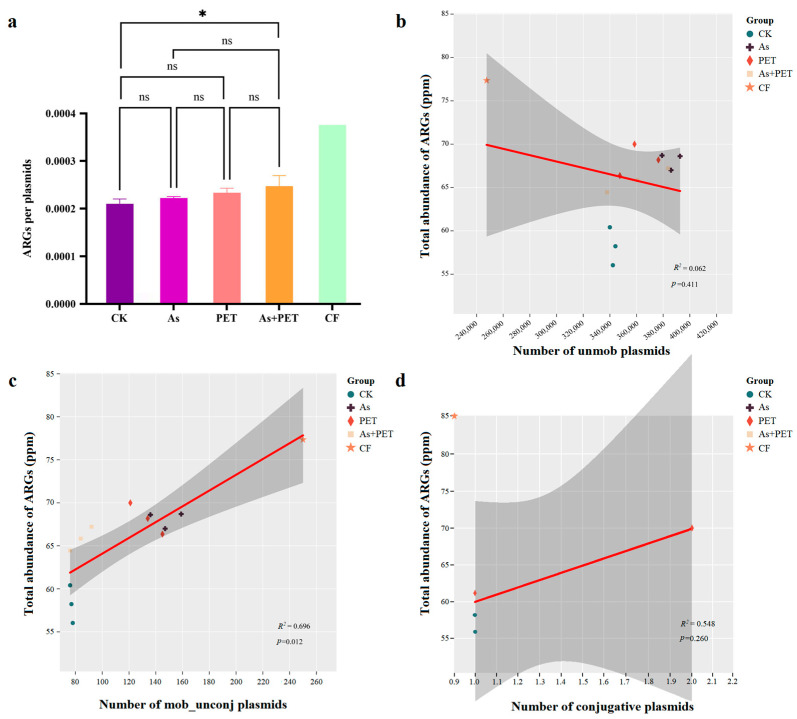
Correlation of ARGs and plasmids. (**a**) ARGs per plasmid among groups (*n* = 3). (**b**–**d**) The correlation between non-mobilizable, mobilizable, and conjugative plasmids and the total abundance of ARGs among groups (*n* = 13). *Symbols indicate statistical significance: * *p* < 0.05, ns represent not significant.

We further analyzed the average number of ARGs carried per plasmid. While the As- and PET-only treatments showed no significant increase in ARG load per plasmid compared to the control, the As + PET co-treatment exhibited a marked rise in both total plasmid abundance and the average number of ARGs per plasmid (Figure 6a).

Heavy metals and microplastics primarily induced a significant increase in mobilizable plasmids. With the exception of a notable increase in non-mobilizable plasmids under heavy metal stress, neither treatment significantly promoted the abundance of non-mobilizable or conjugative plasmids. Statistical analysis revealed a significant positive correlation between mobilizable plasmids and overall ARG variation (*R*^2^ = 0.696, *p* < 0.05), whereas non-mobilizable plasmids showed no such association (Figure 6b–d). Analysis of ARG categories further demonstrated that a broad spectrum of resistance types, including chloramphenicol, MLS, sulfonamide, and tetracycline resistance genes, was significantly associated with mobilizable plasmids (*p* ≤ 0.05; Figure S5).

Additionally, conjugative plasmids were detected in both As- and PET-treated samples: two identical conjugative plasmids were identified under the As treatment, and three were identified under the PET treatment (plasmid profiles are shown in Figure S6). However, no ARGs were annotated on these conjugative plasmids following assembly and functional analyses.

## 3. Discussion

### 3.1. Treatment-Specific Shifts in Plasmid Host Range and Dynamics

The abundance of plasmids varied significantly across treatments, reflecting distinct selective pressures and ecological niches. Vermicomposting alone increased total plasmid abundance, primarily due to a rise in non-mobilizable plasmids, likely because the nutrient-rich earthworm gut promotes bacterial proliferation [5]. Under these conditions, non-mobilizable plasmids may offer a competitive advantage due to their smaller size and lower metabolic cost. In contrast, mobilizable but non-conjugative plasmids declined, possibly due to reduced fitness or transmission benefits in this environment [20].

We acknowledge that the 14-day vermicomposting period in this study is shorter than typical full-scale practices. However, this duration was appropriate under our intensive laboratory conditions (e.g., high earthworm density and pre-composted substrate), which promote rapid processing, as demonstrated by Li et al. (2022) [20]. This experimental design was intentionally chosen to capture the initial, highly dynamic phase of plasmid/ARG selection and enrichment, which are critical for understanding the acute responses to environmental stressors like As and microplastics.

The addition of As alone induced a broad-spectrum increase in all plasmid types, likely resulting from strong selective pressure for As-tolerant bacterial hosts [12]. Once enriched, these hosts carry their respective plasmid complements. The interplay between compensatory adaptation and positive selection within the earthworm gut may further stabilize this host-level enrichment, consequently elevating the abundance of all associated plasmid categories [21]. In contrast, PET microplastics specifically enriched conjugative and mobilizable plasmids. This selective effect can be attributed to the unique ecological niche formed on microplastic surfaces. As part of the dynamic succession process of microbial communities on microplastic surfaces [22], bacteria harboring conjugative plasmids may participate in colonization through surface attachment. The conjugative pili encoded by these plasmids function as adhesion factors that enhance the colonization stability of their hosts on the surface, thereby supporting the persistent presence of such plasmids within the community [23]. Furthermore, biofilm formation on PET surfaces supports high bacterial density, creating a microenvironment conducive to HGT [15,16], which further enhances the dissemination of conjugative and mobilizable plasmids. Notably, the combined PET+As treatment showed minimal net change in total plasmid abundance compared to the control, indicating interactive rather than antagonistic effects. As adsorption onto PET may reduce dissolved As in the gut, weakening broad selection, but it also creates localized As “hotspots” on plastic surfaces [23]. Thus, plasmid dynamics under the co-exposure treatment reflect a balance between weakened bulk-phase selection and intense selection within the plastisphere.

### 3.2. Underlying Mechanisms of Plasmid-Mediated ARG Enrichment Under Distinct Treatments

The mechanisms of ARG enrichment differ under varying pollutant stresses. Arsenic broadly enriched plasmid-associated ARGs through co-selection and enhanced horizontal gene transfer. We observed significant enrichment of arsenic resistance genes (e.g., *arsA*, *arsC*, and *arsR*) under As exposure (Table S2). Given the established co-localization of ARGs and As resistance operons on plasmids [24,25], the concurrent enrichment of both ARGs (e.g., *bla_ampC_*, *bla_FEZ-1_*, *vanS*, and *tetX*) and As resistance genes in this study provides indirect evidence for a co-selection process (Figure S7). Additionally, As stress can trigger oxidative damage and the SOS response [26], which upregulates HGT-related elements and may further facilitate ARG acquisition and dissemination, thereby increasing plasmid-mediated ARG abundance under As stress.

In contrast to the broad pressure exerted by As, microplastics appear to shape the resistome via a “metabolism-resistance” coupling strategy in the plastisphere. In the co-occurrence network, PET-degrading enzymes occupied central positions and were significantly positively correlated with multiple ARGs, particularly multidrug efflux pumps and target modification genes (Figure S7), suggesting an ecological advantage for bacteria harboring these enzymes. The oxidative stress induced during PET degradation may activate the SOS response, thereby upregulating conjugative transfer [27]. Simultaneously, hydrolysis-released monomers provide additional carbon sources that promote biofilm formation, creating an ideal niche for HGT [22]. These conditions may establish a positive feedback loop, enabling PET-degrading bacteria to act as ARG “super-spreaders.” Furthermore, PET’s strong adsorption affinity for antibiotics (e.g., tetracyclines) generates localized high-concentration microenvironments [28,29], which could select for bacteria with efficient efflux pumps. The co-occurrence of the efflux pump gene *mexD* with key esterases and lipases in the same network module supports this “degradation-efflux” co-evolution pattern.

Based on our qualitative observations and the established literature, we propose the following mechanistic hypothesis for the combined PET and As treatment: microplastics function as As carriers, forming localized “high-stress contamination hotspots” on their surfaces [23]. Only bacteria equipped with the most efficient multidrug-resistant systems—such as those carrying broad-spectrum β-lactamases and powerful efflux pumps—could survive in these microenvironments. Although microplastics reduced the total As load in the gut [21], the residual, locally concentrated As maintained sustained selective pressure. This was evidenced by the higher abundance of As resistance genes in the combined treatment compared to the control and As-alone treatments (Table S3). We hypothesize that the synergy between intense local selection and a biofilm platform that enhances HGT frequency transforms the PET+As microenvironment into an ARG “super-mixer,” thereby potentially facilitating the emergence and enrichment of hosts carrying multidrug-resistant plasmids.

### 3.3. The Pivotal Role of Mobilizable Plasmids in ARG Dissemination

Mantel test results demonstrated the dominant role of plasmids in shaping the resistome under the investigated stress conditions. Further analysis revealed a significant correlation between ARG patterns and specific plasmid types, highlighting the crucial contribution of mobilizable but non-conjugative plasmids to ARG variation. Although these plasmids lack a complete conjugation apparatus, they can be trans-mobilized by helper plasmids, thereby substantially promoting ARG dissemination. Statistical analysis confirmed significant correlations with resistance genes encoding chloramphenicol, MLS, sulfonamide, and tetracycline resistance (*p* ≤ 0.05; Figure S5), further confirming their role as key vectors for ARGs under stressful conditions.

In individual As and PET treatments, the absence of a significant increase in ARG load per plasmid indicates that ARG enrichment was mainly attributable to plasmid proliferation rather than the acquisition of additional ARGs per plasmid. In contrast, the As+PET co-treatment significantly increased both the total plasmid count and the average number of ARGs per plasmid. This pattern suggests the integration and optimization of diverse ARGs on plasmids, likely resulting from the stringent multi-resistance pressure within the unique microenvironment created by As adsorption onto microplastics.

Although no ARGs were annotated on the detected conjugative plasmids following assembly and annotation, these elements should still be considered potential risk factors. These “empty” conjugative plasmids can function as highly efficient gene transfer platforms, with the capacity to rapidly acquire ARGs from the environment and disseminate them to pathogenic bacteria under favorable conditions [30].

### 3.4. Implications for Antibiotic Resistance Dissemination Under Pollutant Synergy

Based on the integrated findings of this study, plasmid metagenomic analysis confirms that co-contamination with heavy metals (As) and microplastics (PET) significantly exacerbates the dissemination risk of antibiotic resistance in vermicomposting systems, a critical interface between livestock manure and agricultural soil. The increased diversity of ARGs, coupled with the enhanced mobility and broadened host range of plasmids, indicates that these pollutants not only merely expand the reservoir of ARGs but also actively facilitate their horizontal transfer. The synergistic effect observed under co-treatment conditions is particularly concerning, indicating that in real-world contamination scenarios, resistance risks may be multiplicative rather than merely additive. Microplastics, acting as persistent vectors and hotspots for both contaminants and bacteria [31], can prolong and spatially extend this intensified selective pressure. Consequently, applying such contaminated compost to agricultural fields not only introduces excessive ARGs and resistant bacteria into soil ecosystems but also promotes the horizontal transfer of these genes [32]. By creating a transmission bridge between environmental resistance determinants and clinical pathogens, this process poses substantial long-term threats to soil health, food safety, and public health.

To address this critical challenge, implementing multi-faceted strategies is essential. Primarily, source control must be strengthened through enhanced monitoring and regulation of heavy metals and microplastics in livestock manure, alongside promoting standardized antibiotic use in animal husbandry to reduce pollutant input at the origin. Secondly, in process management, exploring the addition of immobilizing agents or specific microbial consortia to suppress the amplification and transfer of ARGs or optimizing vermicomposting conditions to mitigate pollutant synergy is warranted. Furthermore, for risk early-warning and policy formulation, an environmental safety assessment framework based on the transmission potential of ARGs should be established, integrating the diffusion risk driven by combined pollution into the regulatory standards for compost application in agriculture.

### 3.5. Limitations

In this study, we employed plasmidome metagenomic analysis to investigate the responses of plasmids and their carried ARGs to individual and combined exposures of As and PET. Our results demonstrate distinct effects of these pollutants on the plasmidome and associated ARG profiles. However, several limitations should be considered when interpreting these findings. First, although our sample size met the minimum requirements for statistical analysis, its relatively small scale may limit the generalizability of our conclusions. We therefore recommend that future studies validate these findings with expanded sample sizes to enhance the robustness of the observations. Second, it should be noted that the plasmid DNA enrichment method employed in this study preferentially captures small, high-copy-number plasmids while exhibiting lower extraction efficiency for larger plasmids. This technical bias may lead to an overrepresentation of typically smaller non-mobilizable plasmids and an underestimation of generally larger, low-copy-number conjugative plasmids, consequently affecting our assessment of plasmid-mediated ARG transmission risks [33]. Third, the short-read sequencing technology employed here presents inherent limitations in resolving repetitive regions, assembling complete plasmid sequences, and distinguishing closely related plasmid variants. Additionally, the current lack of specifically optimized tools for plasmid analysis necessitates reliance on indirect read-counting methods that lack standardized protocols for abundance quantification. Future research integrating long-read sequencing technologies with complementary plasmid analysis tools for cross-validation, along with developing standardized quantitative methods, will substantially advance the accuracy and reliability of plasmid studies. Despite these limitations, our study provides valuable insights into plasmidome dynamics and the associated ARG responses, illuminating ARG transmission and evolution in vermicomposting environments.

## 4. Materials and Methods

### 4.1. Materials

The earthworm species *Eisenia fetida* was employed in this experiment, and the earthworms were procured from Tianjin Baiming Technology Development Co., Ltd. (Tianjin, China). Sodium arsenate dodecahydrate (Na_3_AsO_4_·12H_2_O) was purchased from Shanghai Chemical Reagent Procurement Station (Shanghai, China). Polyethylene terephthalate (PET), one of the most commonly used and mass-produced plastics in daily life [34], was selected as the representative microplastic material. The PET (300 μm particle size) samples were sourced from Dongguan Haobang Plastic Chemical Co., Ltd. (Dongguan, China). Prior to their use, the PET microplastics were washed with methanol to remove surface-soluble chemicals, dried at 50 °C, and stored at 4 °C for subsequent experiments. Dairy cattle manure was collected from a dairy farm in Tianjin, China. To enhance the palatability of the manure for earthworms, it was subjected to a 30-day natural composting process for maturation before initiating the experiment.

### 4.2. Experimental Design

An untreated manure control group (CF) was established along with twelve parallel vermicomposting reactors. These reactors were randomly divided into four treatment groups with three replicates each: the CK group (no additives), the As group (As added at 50 mg/kg), the PET group (0.5% dry weight PET microplastics), and the As+PET group (co-exposure to 50 mg/kg As and 0.5% dry weight PET microplastics). The As concentration (50 mg/kg) was selected based on our previous study, which systematically evaluated the responses of ARGs and associated microorganisms to arsenic during dairy manure vermicomposting [20]. The PET microplastic concentration (0.5% dry weight) was determined according to investigation reports on current microplastic pollution levels in agricultural soils [35,36,37]. For As treatment, Na_3_AsO_4_ was dissolved in deionized water and uniformly applied to the manure via spraying while stirring continuously. For the PET treatment, predetermined amounts of PET microplastics were directly incorporated into the manure and thoroughly mixed. Each reactor was loaded with 1.0 kg of pre-composted dairy manure. The reactors utilized vermiculture containers (28 cm × 19.5 cm × 11.5 cm) with perforated bottoms to drain excess leachate, and the tops were covered with gauze to prevent earthworm escape.

**Figure 7:**
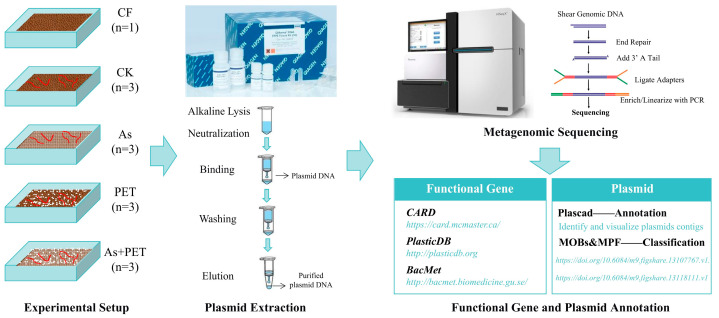
The experimental design and sample handling procedures in this study.

Approximately 24 h prior to the exposure experiment, earthworms were removed from the culture substrate, rinsed with water, and placed on moist filter paper in a dark environment at 20 °C to clear their gut contents and obtain fresh excretions. After this gut-purging process, 300 *Eisenia fetida* worms with similar weights and well-developed clitella were introduced into each vermicomposting reactor. The experiment was conducted under room-temperature conditions (22–24 °C), with distilled water sprayed every three days to maintain the moisture content at 65–75%. Samples were collected upon completion of the vermicomposting treatment, which lasted for 14 days [20]. The experimental period lasted 14 days. Subsequently, manure samples from the untreated control group and earthworm feces from each vermicomposting reactor group were collected for analysis. The experimental design and sample handling procedures are schematically depicted in Figure 7.

### 4.3. Plasmid DNA Extraction from Various Samples

Plasmid DNA was extracted from manure samples collected from all experimental groups using the QIAGEN Plasmid Midi Kit (QIAGEN, Hilden, Germany) according to the manufacturer’s instructions. Three biological replicates were analyzed for each treatment group (CK, As, PET, and As+PET), while a single replicate was processed for the untreated control (CF) group. The obtained plasmid DNA samples were resuspended in DNase-/RNase-free ultrapure water, and their concentrations were quantified using a Qubit 2.0 Fluorometer (Life Technologies, Carlsbad, CA, USA). All DNA extracts were stored at −20 °C prior to subsequent analyses.

### 4.4. Annotation and Quantification of ARGs, MRGs, and Plastic-Degrading Genes

Sequencing was conducted by Shanghai Majorbio Bio-Pharm Technology Co., Ltd. (Shanghai, China) using the Illumina NovaSeq™ X Plu. Each sample obtained over 10 Gb of raw bases. The raw reads from metagenomic sequencing were used to generate clean reads by removing adapter sequences, trimming, and filtering out low-quality reads (reads with N bases, with a minimum length threshold of 50 bp, and a minimum quality threshold of 20) using fastp (version 0.20.0). These high-quality reads were then translated into amino acid sequences using the NCBI translation table. To reduce redundancy, a non-redundant gene catalog was constructed using CD-HIT (version 4.6.1) with the following parameters: 90% sequence identity and 90% coverage. After quality control, sequencing reads were aligned to the non-redundant gene catalog using SOAPaligner (version 2.21) with a stringent 95% identity threshold. The Comprehensive Antibiotic Resistance Database (CARD; https://card.mcmaster.ca/), the antibacterial biocide and MRG database (BacMet; http://bacmet.biomedicine.gu.se/), and the Plastic Biodegradation Database (PlasticDB; http://plasticdb.org) were used to annotate ARGs, MRGs, and proteins linked to plastic biodegradation, respectively. To normalize for variations in sequencing depth and gene length, the abundance of genes is expressed in parts per million (PPM), calculated as (number of reads aligned to the target gene/total number of sequenced reads) × 1,000,000. This normalization procedure eliminates the impact of differences in sequencing throughput across samples and ensures that the abundance values are comparable both within and between samples.

### 4.5. Plasmid Annotation and Classification

High-quality reads were then assembled into contigs using MEGAHIT (version 1.1.2). Contigs with a length of ≥500 bp were selected as the final assembly result [38]. Plascad was employed to identify and visualize plasmids contigs. Plasmid classification was performed based on relaxase types (MOBs) and mating pair formation (MPF) systems. For relaxase-based classification, eight families of N-terminal relaxase protein sequences (~300 amino acids) were used as queries for PSI-BLAST (version 2.17.0) analysis. These reference sequences are available at https://doi.org/10.6084/m9.figshare.13107767.v1. For T4SS classification, four sets of well-characterized type-specific genes (MPF_F_: TraLEKVCWUcNHD; MPF_G_: p31, p35, p41, p44, p51, p52; MPF_I_: TraIKLMNPQRWY; and MPF_T_: VirB3689) were utilized. All reference sequences for T4SS classification are accessible at https://doi.org/10.6084/m9.figshare.13118111.v1 [3].

### 4.6. Statistical Analysis

Experimental data were preliminarily analyzed using Microsoft Excel 2021. PCoA statistical analysis and plotting were carried out using the R language (version 3.3.1). Co-occurrence networks linking ARGs with plasmid contigs and As resistance genes were constructed using Gephi (v0.10.1) to validate host assignments derived from the voting mechanism. Mental tests were generated using Chiplot (https://www.chiplot.online/) [39]. Correlation results, *t*-tests, and the remaining graphs were all analyzed and generated using Origin (version 2024b). Statistical comparisons between groups were performed using either one-way analysis of variance (ANOVA) followed by Turkey’s post hoc test or Student’s *t*-test. Significant differences between groups are defined as * *p* < 0.05 and ** *p* < 0.01. All data are presented as the means ± SEM, and error bars are shown in all figures.

## 5. Conclusions

This study, through a plasmid-centric approach, elucidates the dynamics of plasmid-associated ARGs during vermicomposting of dairy manure under the combined stress of As and PET microplastics. The main findings are as follows:

(1) Different pollutants exert distinct effects on plasmid dynamics during vermicomposting. The As treatment significantly increased the abundance of all plasmid types (non-mobilizable, mobilizable, and conjugative), while PET microplastic exposure selectively promoted the proliferation of mobilizable and conjugative plasmids.
(2) Exposure to pollutants significantly enhanced the enrichment of plasmid-associated ARGs. The As treatment broadly increased the abundance of 12 major ARG types, whereas the PET treatment selectively enriched multiple ARG categories. Notably, although the combined As and PET exposure did not further increase total plasmid abundance, it induced a synergistic enrichment of plasmid-borne ARGs.
(3) Mobilizable plasmids emerged as key vectors for ARG dissemination. A significant positive correlation was observed between the abundance of mobilizable plasmids and total ARG levels, while no such correlation was found for non-mobilizable plasmids. This highlights the specific role of mobilizable plasmids in driving resistance propagation under pollutant stress.

## Data Availability

The metagenomic data of this study are deposited in NCBI under the accession number PRJNA1367325. All other data supporting the results are included within the article and Supplementary Materials.

## Supplementary Materials

The following supporting information can be downloaded at https://www.mdpi.com/article/10.3390/antibiotics14121230/s1.

## Abbreviations

The following abbreviations are used in this manuscript:

ARG	antibiotic resistance gene
MGE	mobile genetic element
MRG	metal resistance gene
HGT	horizontal gene transfer
PET	polyethylene terephthalate
