# Supplementary material for: Plasmid-Mediated Spread of Antibiotic Resistance by Arsenic and Microplastics During Vermicomposting
Source: Antibiotics (Basel). 2025 Dec 6;14(12):1230. doi: 10.3390/antibiotics14121230 (PMC12729771; doi:10.3390/antibiotics14121230)
Supplement: Supplementary file 1 [file antibiotics-14-01230-s001.zip › supporting information.pdf]

Supporting information

Of

Plasmidome variation and Co-selection: Unveiling the Synergistic Mechanism of Arsenic and Microplastics in Promoting Antibiotic Resistance Gene Spread during Vermicomposting

Rui Xin [1, #], Huai Lin[2, #], Zijun Li [1, 3], Fengxia Yang [1, *]

[1] Agro-Environmental Protection Institute, Ministry of Agriculture and Rural Affairs, Tianjin, China;

[2] College of Environmental Science and Engineering, Ministry of Education Key Laboratory of Pollution Processes and Environmental Criteria, Nankai University, Tianjin, China

[3] Chaoyang District Agricultural and Rural Comprehensive Service Center of Beijing, China

[#] These authors contributed equally to this work.

* Correspondence: yangfengxiacomeon@163.com

Figures

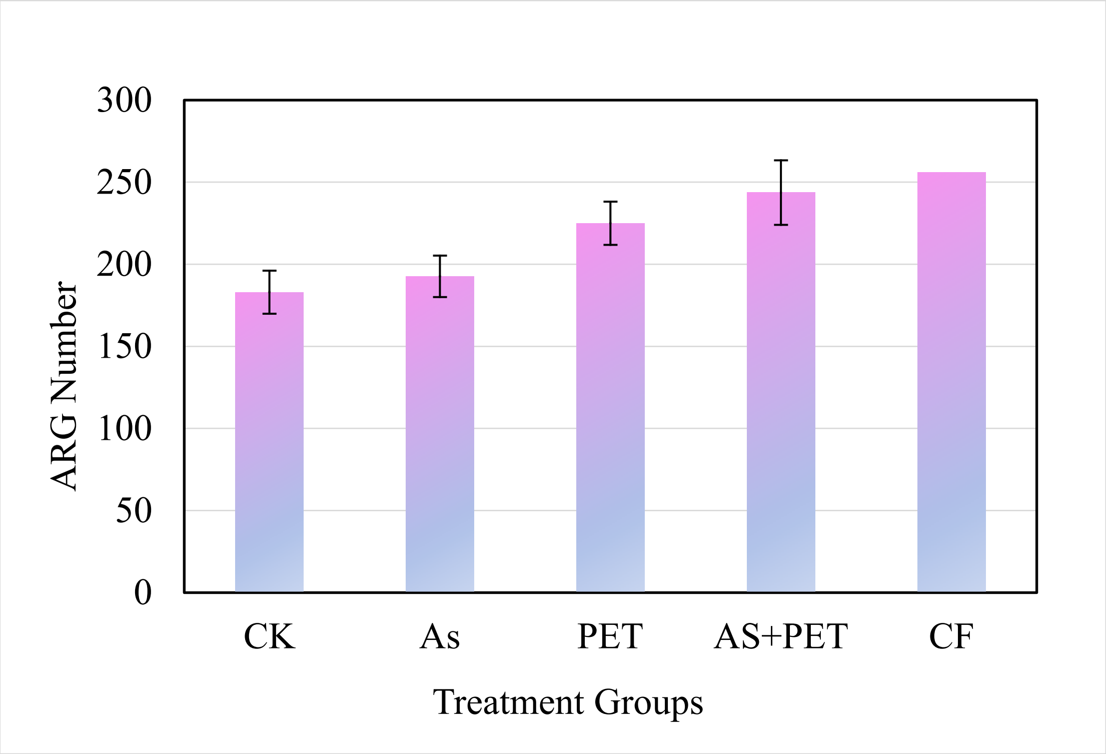

Figure S1. Variations in ARG numbers among the different treatment groups

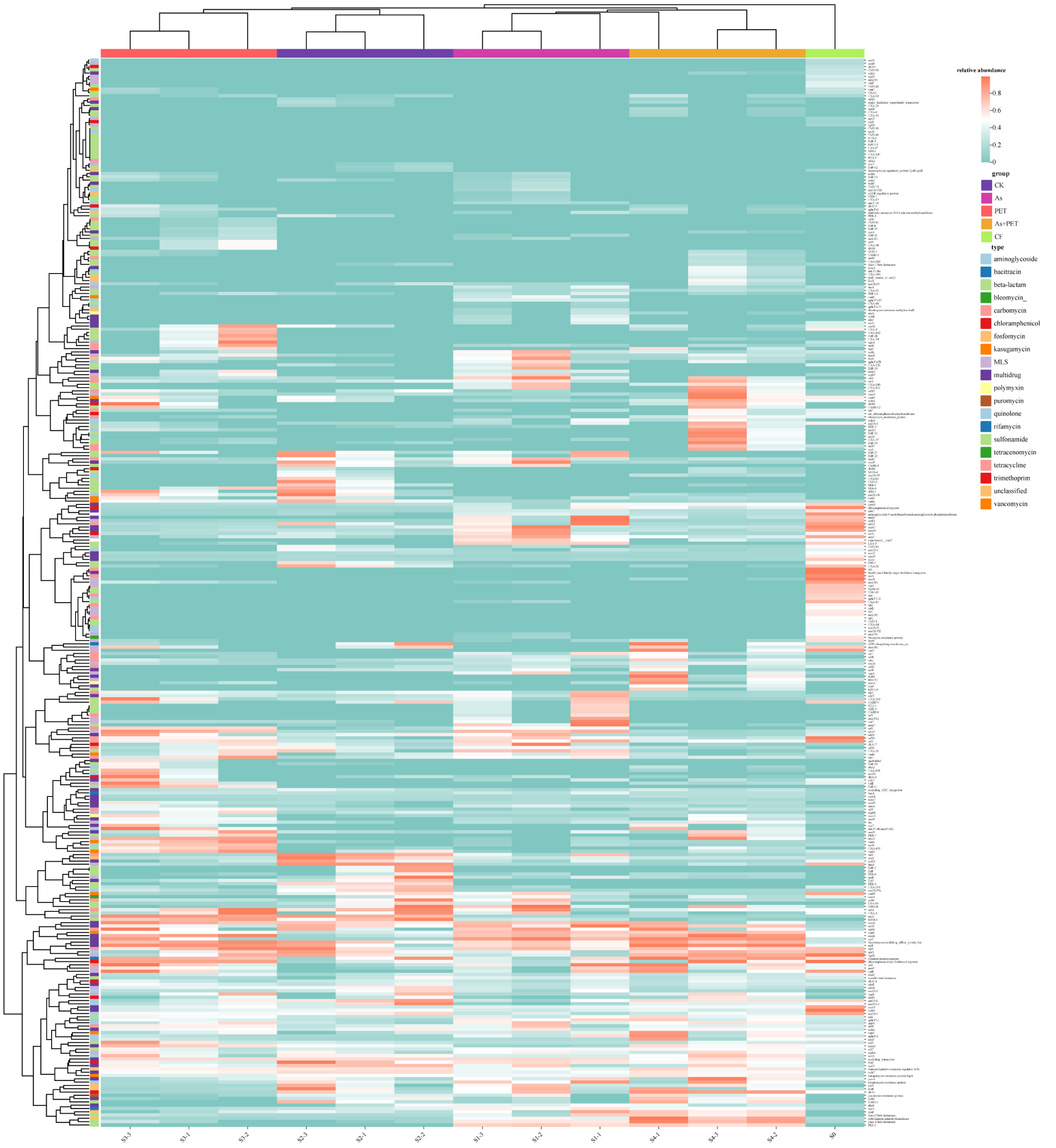

Figure S2. heatmap of ARGs abundance among different treatment groups

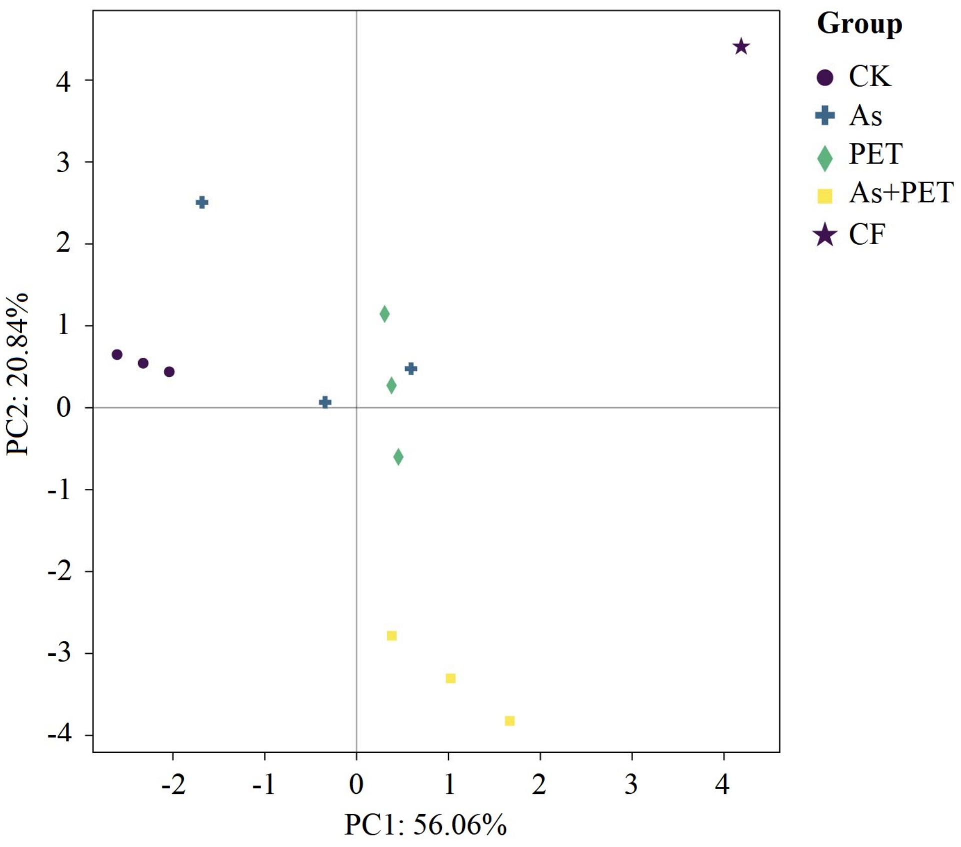

Figure S3. PCA plot of ARGs in different treatment groups.

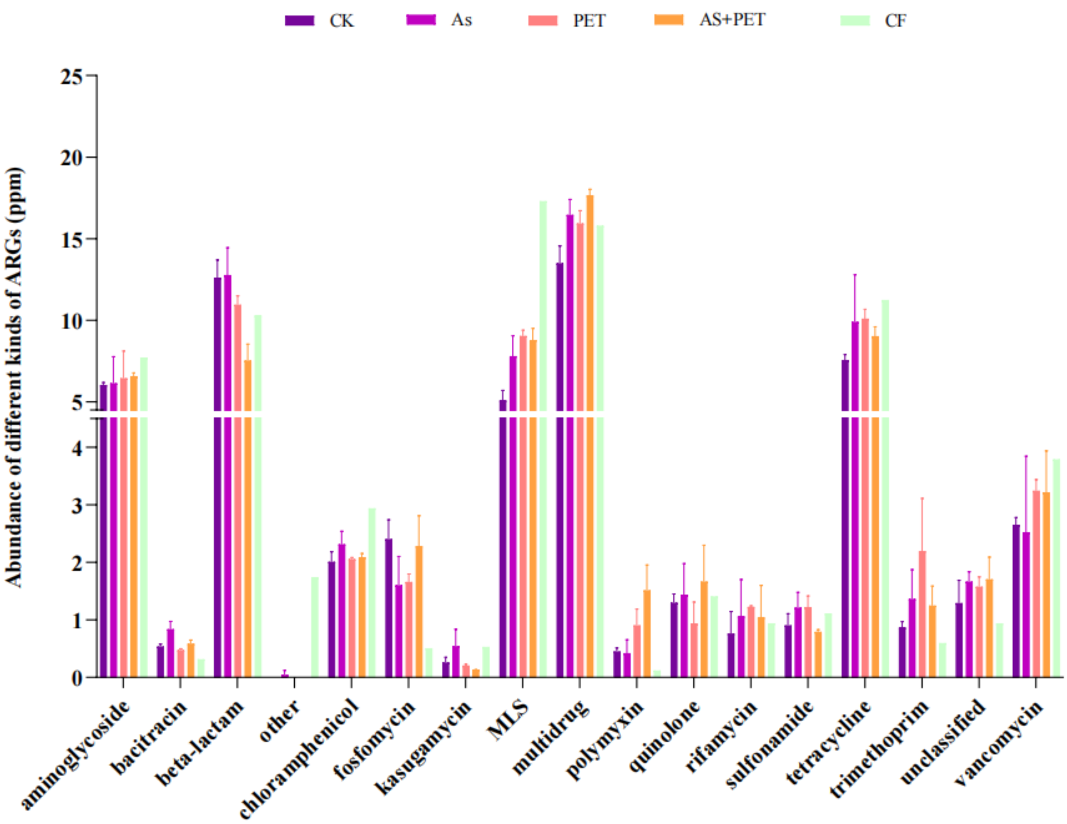

Figure S4. The abundance of ARG types in different treatment groups.

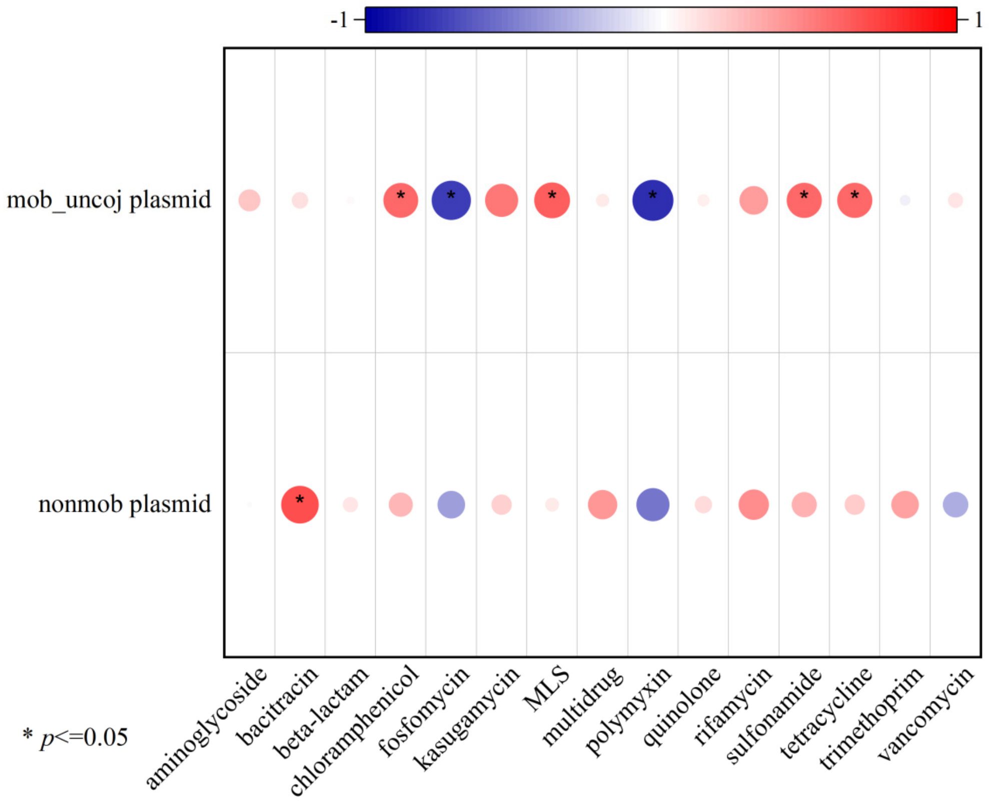

Figure S5. Correlation between plasmid (nonmobilizable plasmids, mobilizable but non-conjugative plasmids) and ARGs

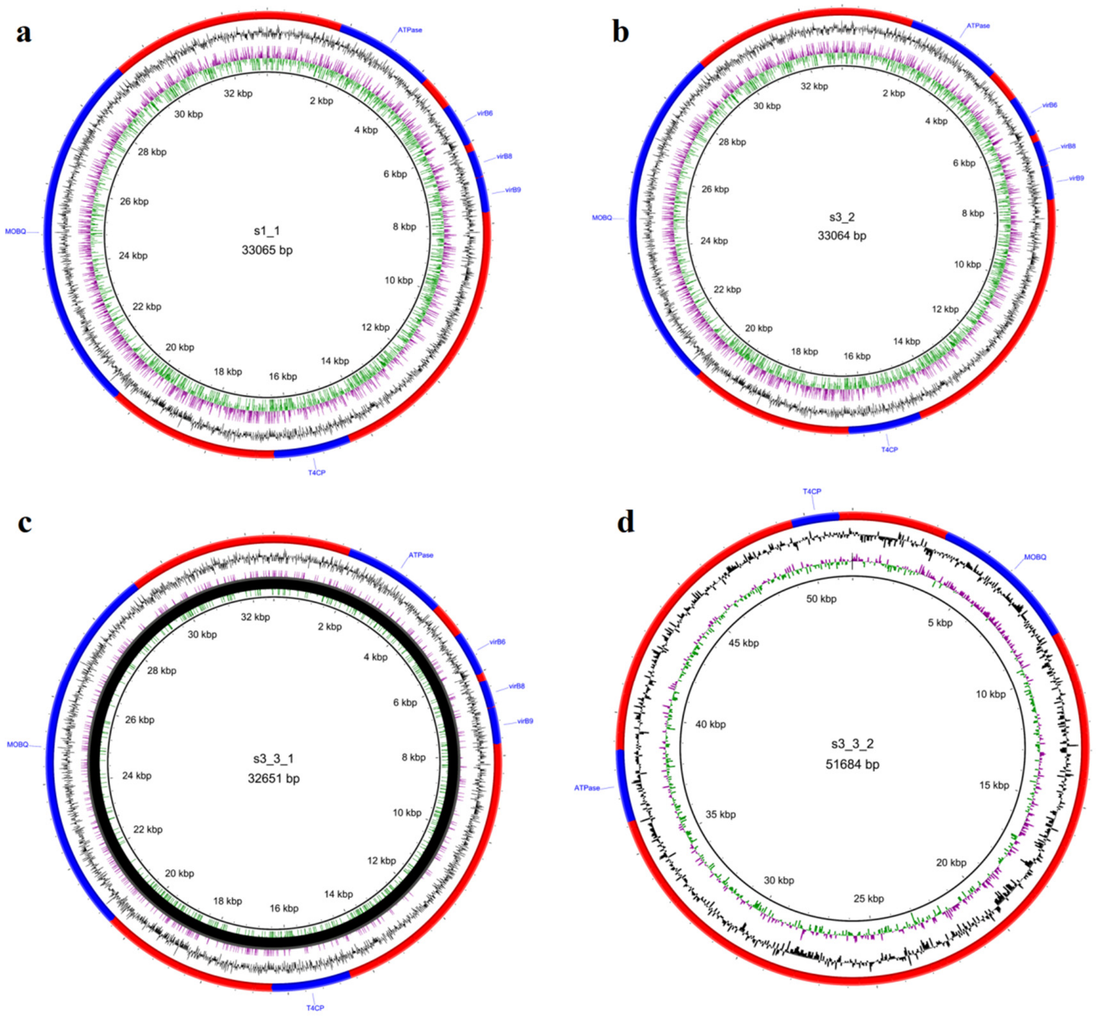

Figure S6. Profiles of conjugative plasmids. (a) Identical plasmid profile of the two conjugative plasmids detected in the As treatment group. (b-d) Profiles of the three distinct conjugative plasmids identified in the PET treatment group.

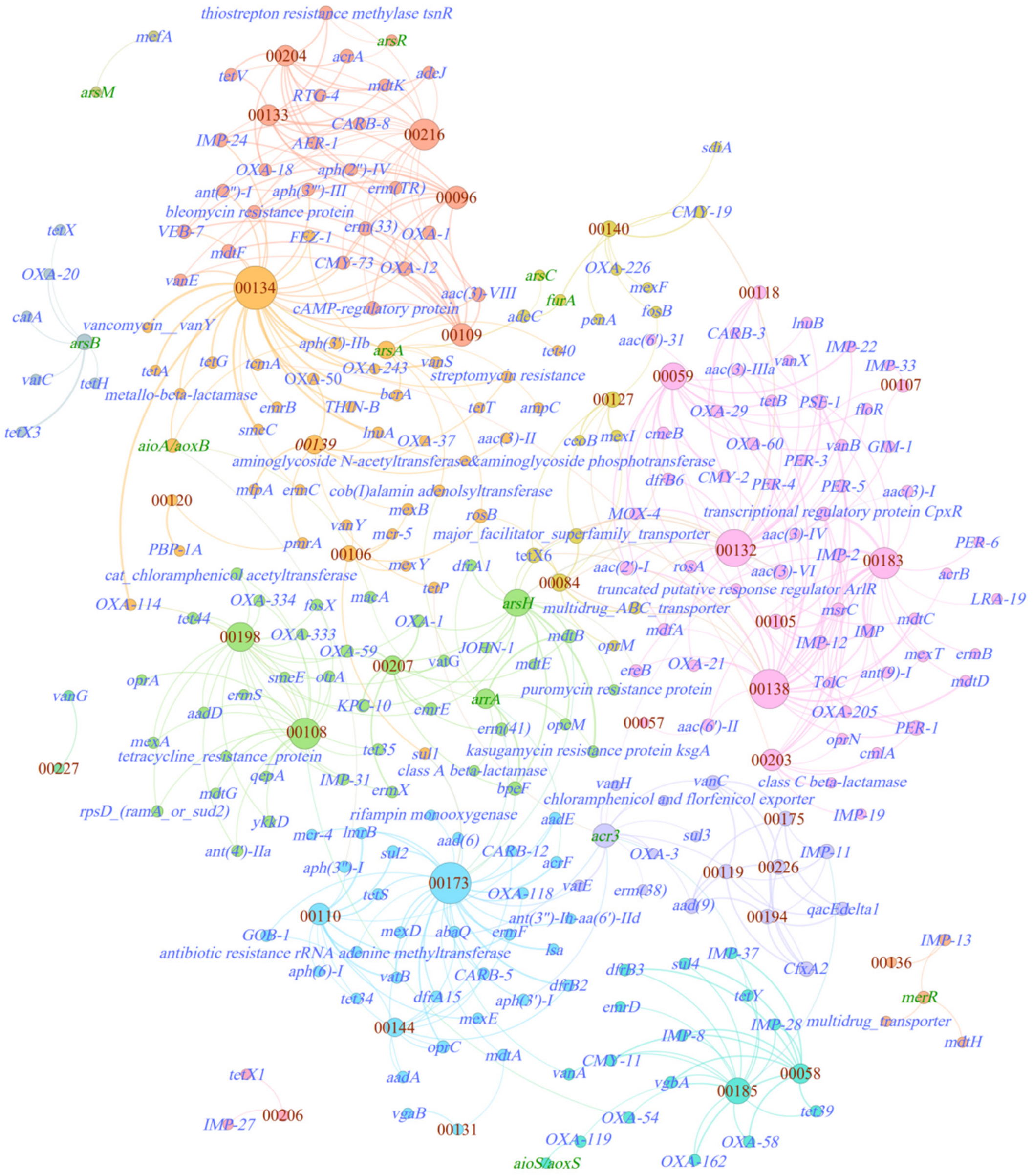

Figure S7. Co-occurrence network between ARGs, arsenic resistance genes and PET-degrading enzymes. Nodes represent ARG/As resistance gene/PET-degrading enzyme and are colored by module membership. Node labels are colored by functional category (blue: ARGs, green: arsenic resistance genes, red: PET-degrading enzymes). Connections (edges) between nodes represent positive and statistically significant ($p < 0.05$) correlations. The size of each node is proportional to its relative abundance.

Tables

Table S2. The abundance of arsenic resistance gene

	As resistance gene abundance (ppm)													
	acr3	*aioA/ aoxB*	*aioE*	*aioR/ aoxR*	*aioS/ aoxS*	*arrA*	*arsA*	*arsB*	*arsC*	*arsH*	*arsM*	*arsR*	*furA*	*merR*
CK	1.36 ± 0.16	0.02 ± 0.02	0.04 ± 0.03	0.20 ± 0.13	0.01 ± 0.01	0.00 ± 0.00	0.76 ± 0.12	3.39 ± 0.58	1.38 ± 0.84	7.42 ± 0.55	1.98 ± 0.32	0.06 ± 0.06	0.09 ± 0.05	0.78 ± 0.09
AS	1.18 ± 0.09	0.09 ± 0.06	0.07 ± 0.06	0.28 ± 0.17	0.00 ± 0.00	0.00 ± 0.00	1.04 ± 0.29	3.51 ± 0.29	1.98 ± 0.93	6.63 ± 0.86	2.16 ± 0.31	0.10 ± 0.08	0.11 ± 0.04	0.89 ± 0.18
PET	1.63 ± 0.11	0.05 ± 0.02	0.06 ± 0.06	0.13 ± 0.03	0.02 ± 0.01	0.00 ± 0.00	0.78 ± 0.18	3.43 ± 0.18	1.06 ± 0.21	5.99 ± 0.06	1.70 ± 0.27	0.06 ± 0.02	0.03 ± 0.02	1.03 ± 0.39
AS+ PET	1.54 ± 0.18	0.06 ± 0.02	0.08 ± 0.02	0.31 ± 0.06	0.02 ± 0.04	0.04 ± 0.04	1.06 ± 0.11	3.10 ± 0.22	1.56 ± 0.23	8.76 ± 0.77	1.83 ± 0.36	0.08 ± 0.07	0.12 ± 0.06	0.64 ± 0.02
CF	3.48	0.00	0.04	0.12	0.00	0.00	0.27	7.95	8.90	6.49	5.36	0.64	0.97	0.41

CK were the control group (only added earthworm without pollution exposure), As were the Arsenium treatment (Arsenium exposure after earthworm add), PET were the polyethylene terephthalate treatment (PET exposure after earthworm add), AS+PET were the Arsenium and PET combined treatment (AS+PET exposure after earthworm add) and CF were the untreatment (origin fecal without any added and treatment).

Table S3. The abundance of MRGs

	MRG type abundance (ppm)																
	Ag	Al	As	Au	Cd	Co	Cr	Cu	Fe	Hg	Ni	Pb	Se	Te	V	W	Zn	multimetal
S2-1	8.00	0.44	21.93	3.21	0.02	0.10	17.93	34.90	31.16	4.68	0.25	1.50	0.87	0.92	2.21	0.00	5.53	61.01
S2-2	8.73	0.13	16.02	3.26	0.03	0.14	21.36	28.82	20.36	3.69	0.16	1.21	1.16	0.65	2.40	0.00	7.13	61.97
S2-3	7.18	0.21	17.25	3.23	0.00	0.10	19.77	30.52	20.89	4.77	0.10	1.35	1.36	0.91	2.15	0.00	7.24	56.55
S1-1	8.22	0.80	22.27	3.56	0.10	0.07	18.55	40.04	34.16	4.80	0.39	2.85	0.88	0.73	2.34	0.00	5.20	62.72
S1-2	6.48	0.50	18.64	2.18	0.00	0.08	16.36	31.00	25.43	5.02	0.16	1.84	1.17	0.67	2.14	0.00	5.13	55.76
S1-3	6.58	0.28	15.92	2.99	0.04	0.09	21.19	30.58	18.46	4.71	0.16	1.37	1.09	0.53	2.87	0.00	5.78	55.67
S3-1	7.46	0.25	16.08	2.70	0.03	0.02	24.85	29.02	20.95	5.15	0.05	1.36	1.33	0.75	2.78	0.02	8.34	63.88
S3-2	5.93	0.52	16.10	2.50	0.02	0.07	20.23	29.38	22.79	4.50	0.29	2.78	1.20	0.71	1.96	0.00	6.98	58.01
S3-3	5.58	0.46	18.57	2.46	0.00	0.20	18.46	32.84	23.76	4.66	0.17	1.66	1.64	0.68	2.29	0.00	6.75	61.06
S4-1	8.55	0.40	20.18	2.68	0.07	0.00	19.80	36.80	27.44	3.28	0.15	1.42	1.21	0.53	1.99	0.00	7.42	69.75
S4-2	7.95	0.41	18.75	2.45	0.02	0.02	17.87	31.93	26.54	4.51	0.30	1.63	1.38	0.52	1.98	0.00	6.12	61.09
S4-3	7.78	0.56	19.47	3.22	0.04	0.03	18.38	36.35	32.53	3.87	0.28	1.45	0.89	0.48	1.82	0.00	5.42	67.97
S0	2.55	1.81	45.82	1.32	0.12	0.21	19.74	33.17	80.58	5.67	0.27	1.38	1.32	0.66	0.70	0.08	2.84	57.25
